# Supplementary material for: A UMLS-based spell checker for natural language processing in vaccine safety
Source: BMC Med Inform Decis Mak. 2007 Feb 12;7:3. doi: 10.1186/1472-6947-7-3 (PMC1805499; doi:10.1186/1472-6947-7-3)
Supplement: Additional file 13 — RAP application directory. Sets up directory for RAP (RDF application for PHP) source code [file 1472-6947-7-3-S13.gz › rap/doc/phpdoc/infModel/InfStatement.html]

xml version="1.0" encoding="iso-8859-1"?


Docs For Class InfStatement


## Class InfStatement

Description

Description |
Methods

An RDF statement which was entailed by a inference rule.

See Statement Class for further information.

History:

- version: V0.9.1
- author: Daniel Westphal <at d-westphal dot de mail at d-westphal dot de>

Located in /infModel/InfStatement.php (line 17)

```
Object
   |
   --Statement
      |
      --InfStatement
```

Methods

Description |
Methods

#### Inherited Methods


Inherited From Statement

> Statement::Statement()  
> Statement::compare()  
> Statement::equals()  
> Statement::getLabelObject()  
> Statement::getLabelPredicate()  
> Statement::getLabelSubject()  
> Statement::getObject()  
> Statement::getPredicate()  
> Statement::getSubject()  
> Statement::hashCode()  
> Statement::object()  
> Statement::predicate()  
> Statement::reify()  
> Statement::subject()  
> Statement::toString()  
> Statement::toStringObject()  
> Statement::toStringPredicate()  
> Statement::toStringSubject()

Inherited From Object

> Object::toString()

Documentation generated on Thu, 7 Jul 2005 13:41:45 +0200 by phpDocumentor 1.3.0RC3
